# Accession-specific parent-of-origin dependent and independent genome dosage effects on salt tolerance in *Arabidopsis thaliana*

**DOI:** 10.1098/rsos.231766

**Published:** 2024-05-08

**Authors:** Brendan F. Hallahan, Luis Felipe Quiroz, Galina Brychkova, Peter C. McKeown, Charles Spillane

**Affiliations:** ^1^ Agriculture and Bioeconomy Research Centre, Ryan Institute, University of Galway, Galway, H91 REW4 , Ireland

**Keywords:** genome dosage, abiotic stress, epigenetic, parent-of-origin, *Arabidopsis*

## Abstract

Improving the salt stress tolerance of crops is an important goal in plant breeding. Changes in the number of chromosome sets (i.e. ploidy level) cause genome dosage effects which can result in enhanced or novel traits. Maternal inheritance versus paternal inheritance of the same chromosome sets can have differential epigenetic effects on traits of F1 offspring. Hence, genome dosage effects can be parent-of-origin independent or dependent. The model plant *Arabidopsis thaliana* displays both genome dosage and parent-of-origin effects on plant growth under non-stress conditions. Using an isogenic ploidy series of diploid, triploid and tetraploid lines, we investigate the extent of genome dosage effects and their parent-of-origin dependency on *in vitro* salt stress tolerance of seedlings across 10 different *A. thaliana* accessions (genetic backgrounds). We detected genome dosage effects on salt stress tolerance for tetraploid lines in five accessions. In addition, through the generation of isogenic reciprocal F1 triploid lines, both parent-of-origin dependent and independent genome dosage effects on salt stress tolerance were detected. Thus, our results indicate not only that genome dosage balance effects can have significant impacts on abiotic stress tolerance in *A. thaliana* but also that parent-of-origin specific genome dosage effects can affect salt stress tolerance in plants.

## 1. Introduction

Polyploidy is the phenomenon where an organism possesses more than two sets of chromosomes per cell nucleus. Polyploidy can result from whole genome duplication events, which are a major mechanism of plant evolution and speciation [1–3]. When polyploids form, their increased genome dosage can lead to the emergence of new or enhanced traits [4,5]. Genetic redundancy within polyploid genomes can allow duplicated genes to take on a new function (neofunctionalization) or retain different components of an original function (subfunctionalization) [2,3,6,7].

Gene and genome dosage effects on plant growth have been reported in allopolyploids (polyploids with divergent genomes) [8,9]. However, allopolyploids are (by definition) genetic hybrids, wherein determining the contribution of genome dosage versus genetic hybridity is difficult to disentangle. In contrast, by using autopolyploids (polyploids with genomes of the same type), it is possible to create isogenic ploidy series which differ only in the number of chromosome copies in the nucleus. Autopolyploid research in *Arabidopsis thaliana* [10–13] and maize (*Zea mays*) [14,15] has shown genome dosage effects on plant growth and development that can be either parent-of-origin independent or dependent. The induction of autopolyploidy in commercial crops for improvement in yield and quality has been used in potato (*Solanum tuberosum*) [16], sugarcane (*Saccharum officinarum*) [17], perennial ryegrass (*Lolium perenne*) [18], blueberry (*Vaccinium corymbosum*) [19] and alfalfa (*Medicago sativa*) [20] among other crops. In addition to increasing yield and quality, improving abiotic stress tolerances of crops is an important objective in plant breeding, particularly for soils subject to salinization and saline agricultural systems (i.e. salt stress) [21,22]. However, there has been limited research investigating genome dosage effects on salt stress tolerance [23].

To date, research on the abiotic stress response of polyploid plants has revealed both positive, negative and neutral genome dosage effects. In chrysanthemum (*Chrysanthemum indicum*), for a single genotype, it was demonstrated that cold, salt and drought tolerance were improved upon induction of tetraploidy, but heat tolerance was greater at the diploid level [24]. Using field transplant experiments of wild yarrow (*Achillea borealis*), hexaploid plants are more likely to survive sand dune environments than tetraploid plants, although population effects are also significant [25]. Enhanced drought tolerance of wild willowherb (*Chamerion angustifolium*) at the tetraploid level over the diploid level has been demonstrated [26]. In *A. thaliana*, it has been reported, for a small number of accessions, that plants are more salt tolerant at the tetraploid level than at the diploid level, as measured by days-to-death, seed yield and levels of anthocyanin [27,28]. Taken together, data from diverse plant species across different growing habits suggest that genome dosage effects on abiotic stress response can occur. However, there has been no significant investigation of parent-of-origin dependent genome dosage effects on abiotic stress responses.

Studies on genome dosage effects on abiotic stress responses in plants have a limited focus on salt stress tolerance. In addition, there have been no investigations of the presence or extent of epigenetic parent-of-origin dependent genome dosage effects on salt stress tolerance. Hence, we have used the model plant *A. thaliana* to investigate whether genome dosage effects on salt tolerance occur, and the extent to which such effects could be parent-of-origin dependent or independent. Thus, in this study, we used NaCl as the source of saline stress, where we defined the stress as a major imbalance between the environment and physiology [29], but which did not allow the stress to lead to plant death. We sought to determine if tolerance to salt stress in *A. thaliana* (across a range of genetic backgrounds) was subject to genome dosage effects, and whether any such effects were parent-of-origin dependent or independent. Our results not only reveal the significant impact of genome dosage on salt stress tolerance in *A. thaliana* but also demonstrate that the nature of this effect can be parent-of-origin dependent or independent in an accession-specific manner. Thus, our findings introduce novel perspectives for ploidy-manipulation-based crop breeding practices with potential applications in enhancing salt stress resistance in crop plants.

## 2. Material and methods

### 2.1. Plant material and crossing design

Ten *A. thaliana* accessions were kindly provided at both the diploid and tetraploid levels. C24, Col-0, L*er*-0 and Zu-0 were the kind gift of Luca Comai (UC Davis, CA, USA) and Bur-0, Cvi, Sorbo, T910, TAL07, Wilna were the kind gift of Ortrun Mittelsten Scheid (Gregor Mendel Institute, Vienna, Austria). The country of origin of each accession is as follows: Bur-0, Ireland; C24, Portugal; Col-0 and L*er*-0, Germany; Cvi, Cape Verde Islands; Sorbo, Tajikistan; T910 and TAL07, Sweden; Wilna, Lithuania; Zurich, Switzerland. Plants were grown in 7 × 7 × 6.5 cm^3^ pots (Modiform, Leusden, The Netherlands) in soil (5:1:1 mix of peat:vermiculite:perlite). Growth room (Cambridge HOK, East Yorkshire, UK) conditions were 16/8 h light/dark at 22/20°C. Plants were maintained for at least six generations before crossing. Reciprocal triploids were generated by manually emasculating flowers on lateral stems with Dumont no. 5 tweezers (Electron Microscopy Sciences, PA, USA) and reciprocally crossing the diploid and tetraploid lines in both directions: 2 × ♀ × 4 × ♂ crosses produced paternal-excess triploids—labelled 3×(p), while 4 × ♀ × 2 × ♂ crosses produced maternal-excess triploids— labelled 3×(m). A single maternal and single paternal plant were used for all crosses, except for Col-0 and Zu-0, where three maternal plants were used to generate sufficient 3×(p) F1 seed due to the strong triploid block. Self-pollinated diploid and tetraploid siliques were harvested from the maternal parent on the same lateral stem used for generating triploids.

### 2.2. Growth media

All chemicals were obtained from Sigma Aldrich, Ireland. Growth media were prepared by adding one-half strength Murashige and Skoog basal medium and sucrose at 0.5% (w/v) to distilled water. The required amount of NaCl was added. The solution was brought to pH 5.7 using 1 M KOH dropwise. Lastly, agar at 0.8% (w/v was added before sterilization.

### 2.3. Plant stress experiments

To quantify different levels of salt stress tolerance, we take as a proxy the plant biomass produced in saline conditions versus non-saline conditions [30]. Plants were grown in artificial growth media *in vitro* supplemented with NaCl as follows. Seeds were first surface sterilized with 70% methanol, then a seed sterilization solution consisting of 5% sodium hypochlorite solution (NaClO) with 0.01% (v/v) Triton X-100, followed by five washes in sterile, distilled water. Seeds were stratified for three days in the dark at 4°C. Seeds were sown to fresh, stress-free media in 100 × 100 × 20 mm^3^ Petri dishes (Sarstedt, Nümbrecht, Germany) sealed with Micropore™ tape (3M, MN, USA). Plates were horizontally positioned in a growth chamber (CLF Plant Climatics, Emersacker, Germany) with 16/8 h light/dark at 22/20°C. After 2 days, the plates were positioned vertically. Plants that were 9 days old (growth stage 1.02 [31]) were transferred to fresh media with or without NaCl.

To determine the relative salt stress tolerance of all 10 accessions at their basal ploidy level and identify the optimal salt concentration that did not induce bleaching, 9-day-old diploid plants were grown in media supplemented with either 0, 50, 75, 100, 125 or 150 mM NaCl. Subsequently, to determine genome dosage and parent-of-origin effects on salt stress tolerance, all 10 accessions were investigated at the diploid, tetraploid and reciprocal triploid levels (i.e. an isogenic ploidy series). Diploid and tetraploid plants of the same accession were grown together in the same vertical plates to minimize any plate effect, as were reciprocal triploid plants. Stress plates contained a maximum of five biological replicates per ploidy level while stress-free plates contained a maximum of three biological replicates. After 7 days, plants were destructively harvested for fresh weight measurements: plants were dried on a paper towel and weighed on a NewClassic MF weighing scale (Mettler Toledo, Greifensee, Switzerland) to the ten-thousandth decimal value. The term shoot is used in this study to mean all plant biomass from the hypocotyl upwards, while the term root means all biomass below the hypocotyl. Plants were snipped in two parts (above and below ground) using Dumont no. 5 tweezers. The percentage of plant biomass produced in saline conditions versus non-saline conditions was calculated. Similarities between pooled ploidy fresh weight data were analysed with Pearson’s correlation coefficient. Interaction effects between accumulated fresh weight under saline conditions for ploidy level and accession were analysed with a two-way analysis of variance (ANOVA). Statistical differences for genome dosage and parent-of-origin effects on salt stress tolerance for each accession were determined with a one-way ANOVA followed by Tukey’s honestly significant difference (HSD) test.

### 2.4. Flow cytometry

Plants were grown in a growth room as before. All chemicals were obtained from Sysmex, Kobe, Japan. Approximately, 3 cm^2^ of leaf material from one-month-old plants was removed and chopped with a razor blade in the presence of 400 µl nuclei extraction buffer. After 5 min, the mixture was strained into a 3.5ml Röhren tube through a 30 μm CellTrics^®^ filter. One millilitre of UV stain was added before the sample was analysed on a Partec Ploidy Analyzer (Sysmex, Kobe, Japan). Ploidy levels of diploid, tetraploid and reciprocal triploid plants were confirmed (electronic supplementary material, S1).

## 3. Results

### 3.1. Concentration of 125 mM NaCl induces salt stress in most *A. thaliana* accessions at the diploid level *in vitro*

**Figure 1.**
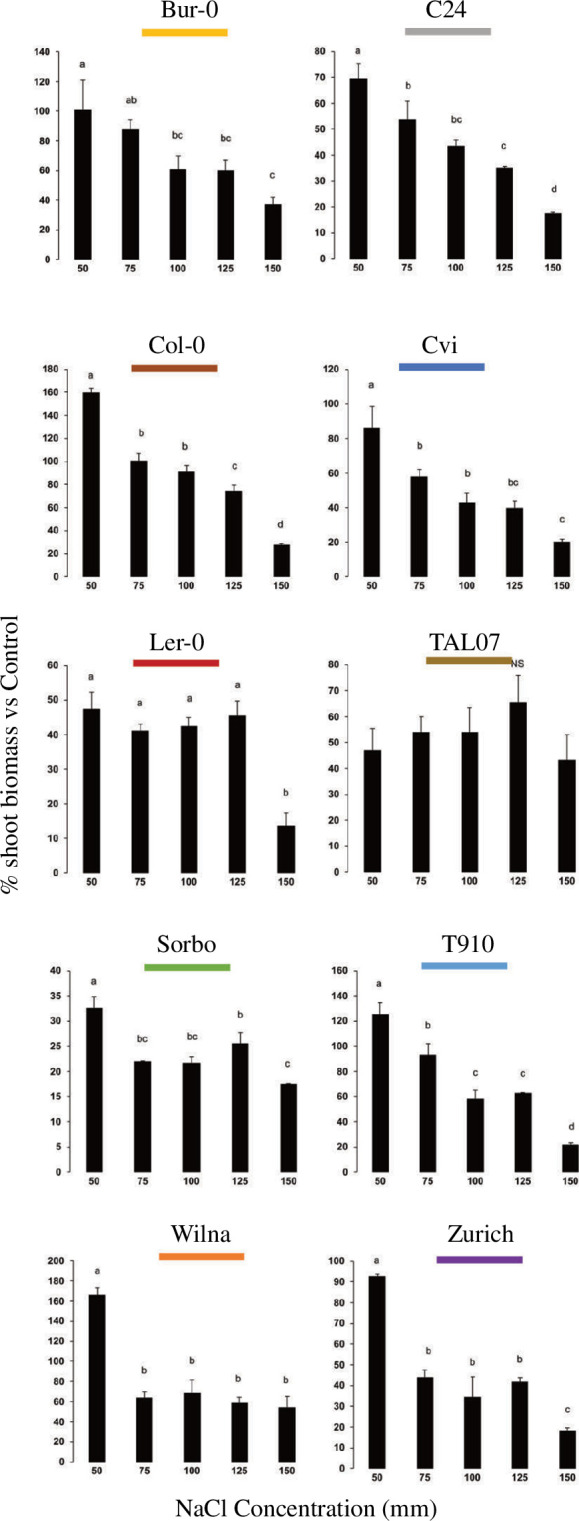
Above-ground biomass accumulation of 10 *A. thaliana* accessions at the diploid level under a range of salt concentrations. Plants at their basal ploidy level (diploid) were germinated in stress-free artificial growth media and after 9 days transferred to fresh media with or without NaCl. Plants were destructively harvested after 7 days. Each accession was analysed with a one-way ANOVA, and means assigned different letters are statistically different (*p* < 0.05) according to Tukey’s HSD test; NS is not statistically different (*p* > 0.05). Error bars represent standard deviation.

To determine the highest salt stress concentration that neither stopped seedling growth nor induced bleaching, all 10 accessions, at their basal ploidy level (diploid, apart from Wilna which also has naturally occurring tetraploid populations), were grown in media supplemented with a range of NaCl concentrations. Using these criteria, it was determined that 125 mM NaCl is optimal to induce salt stress in most accessions. The accession Cvi-0 was identified as particularly salt-sensitive, as previously reported [32], and, thus, we determined should be tested at 100 mM NaCl (figure 1).

### 3.2. Salt stress tolerance across *A. thaliana* ploidy levels is dependent on genetic background

**Figure 2.**
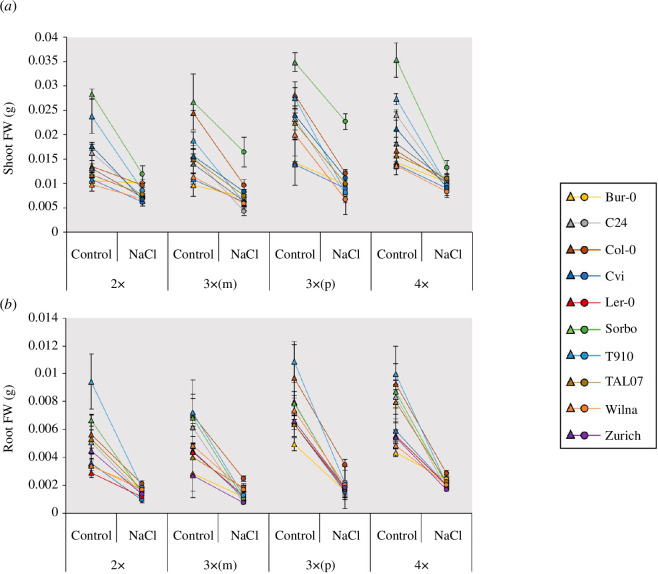
Salt stress causes a reduction in plant biomass across all ploidy levels in *A. thaliana*. Shoot FW (*a*) and Root FW (*b*) of all accessions at four ploidy levels under control and NaCl. Plants were germinated in stress-free artificial growth media and after 9 days transferred to fresh media with or without NaCl. Plants were destructively harvested after 7 days.

All 10 accessions in an isogenic ploidy series were tested under salt stress. It was observed that all ploidy levels experienced some reduction in shoot and root growth under salt stress, albeit to varying levels (figure 2*a*,*b*; electronic supplementary material, tables S1 and S2). Shoot FW under control and salt stress conditions were modestly correlated for diploid (Pearson’s *r* = 0.48) and tetraploid plants (*r* = 0.48), and strongly correlated for maternal-excess triploids (*r* = 0.71) and paternal-excess triploids (*r* = 0.69) (electronic supplementary material, table S3), suggesting some similarities in salt stress tolerance between accessions at each ploidy level. Averaging across genetic backgrounds may hide details, hence an interaction effect between accession and ploidy level under salt stress was also investigated, that is, genome dosage and parent-of-origin salt stress effects may be context-dependent. An interaction plot of shoot and root biomass accumulated in saline conditions versus non-saline conditions and ploidy level for each accession (electronic supplementary material, figure S1) reveals an absence of clear parallel lines, hinting that there could be an interaction phenomenon. To clarify this, we analysed the effects of accession, ploidy level and their interaction in a two-way ANOVA. The interaction of accession × ploidy level on shoot biomass accumulation under salt stress is statistically significant (*F*_27,80_ = 2.84, *p* < 0.001); likewise, the interaction of accession × ploidy level on root biomass accumulation under salt stress is statistically significant (*F*_27,80_ = 4.37, *p* < 0.001) (electronic supplementary material, table S4). This indicates that genome dosage and parent-of-origin dependent and independent genome dosage effects on salt stress tolerance are accession-specific. While some genetic backgrounds may display genome dosage effects on salt stress tolerance, others may not.

### 3.3. Genome dosage effect on salt stress tolerance between 2× and 4× isogenic lines

**Figure 3.**
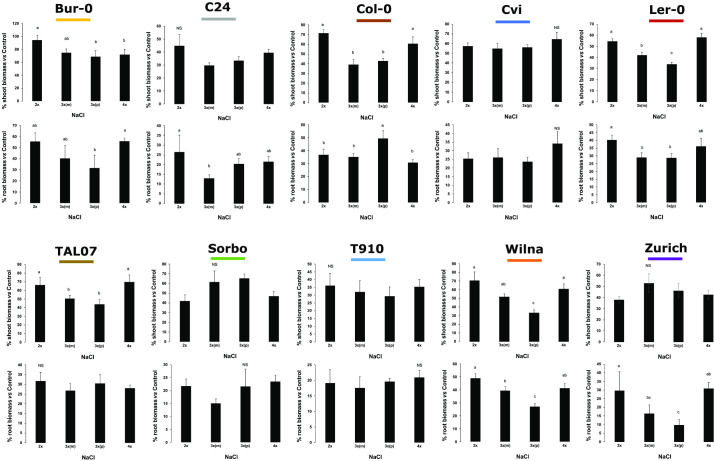
Parent-of-origin dependent and independent genome dosage effects on salt tolerance in *A. thaliana.* There is a parent-of-origin independent genome dosage effect on salt stress tolerance in Bur-0, Col-0, TAL07, Wilna, Zurich and L*er*-0. There is a parent-of-origin dependent genome dosage effect on salt stress tolerance in Col-0, L*er*-0 and Wilna. Plants were germinated in stress-free artificial growth media and after 9 days transferred to fresh media with or without NaCl. Plants were destructively harvested after 7 days. Each accession was analysed with a one-way ANOVA and means assigned different letters are statistically different (*p* < 0.05) according to Tukey’s HSD test; NS is not statistically different (*p* > 0.05). Error bars represent standard deviation.

Comparisons between genetically isogenic autopolyploid diploid and tetraploid plants allow us to identify genome dosage effects in relation to any differential phenotypes. In both NaCl stress and NaCl stress-free media tetraploid plants were larger than diploid plants, as measured for both above (shoot)- and below (root)-ground fresh weight (figure 2; electronic supplementary material, S2). Of the 10 genetic backgrounds (i.e. accessions) analysed, only one accession displays a genome dosage effect difference in salt stress tolerance of shoot biomass between diploid and tetraploid plants. In the accession Bur-0, diploid plants accumulate significantly (*p* < 0.05) more above-ground fresh weight (electronic supplementary material, S2) and showed lower biomass reduction than tetraploid plants under salt stress conditions, that is, possibly more salt stress tolerant (figure 3). For all other accessions, diploid and tetraploid plants showed similar above- and below-ground biomass reduction under salt stress (figure 3). Despite tetraploid plants being larger than diploid plants, on average diploid and tetraploid plant biomass is reduced by approximately 50% and below-ground biomass is reduced by approximately 70% by salt stress (figure 3).

### 3.4. Parent-of-origin independent genome dosage effect on salt stress tolerance

Comparison of the salt tolerance of diploid lines with isogenic triploid counterparts allows for a test of genome dosage effects on salt tolerance. Where such genome dosage effects occur, if both the maternal-excess and paternal-excess triploid lines display an equivalent genome dosage effect this can be considered as a parent-of-origin independent genome dosage effect. There are five cases where both maternal- and paternal-excess triploid plants displayed a higher biomass reduction under salt stress than their diploid equivalent, indicating a parent-of-origin independent genome dosage effect on salt stress tolerance (figure 3). In the cases of Col-0 and TAL07, the above-ground shoot biomass of diploid plants displayed a lower biomass reduction than both maternal- and paternal-excess triploid plants. In the cases of Wilna and Zurich, the below-ground biomass of diploid plants was less affected than both maternal- and paternal-excess triploid plants. In the case of the accession L*er*-0, both the above- and below-ground biomass reduction is significantly lower in diploid plants than in maternal- and paternal-excess triploid plants (figure 3).

Similarly, the comparison of tetraploid lines with their isogenic triploid counterparts also allows for an additional test of genome dosage effects on salt tolerance. We identify three cases where the biomass reduction under salt stress in both maternal- and paternal-excess triploid plants was higher than their tetraploid equivalent, indicative of a parent-of-origin independent genome dosage effect on salt stress tolerance (figure 3). In the cases of Col-0, L*er*-0 and TAL07, tetraploid plant above-ground biomass was less affected by salinity than both maternal- and paternal-excess triploid plants.

### 3.5. Parent-of-origin dependent genome dosage effects on salt stress tolerance

F1 triploid plants generated via reciprocal crosses between genetically isogenic diploid and tetraploid parents can be used to identify parent-of-origin dependent genome dosage effects. Paternal excess F1 triploid plants have nuclei containing two paternally inherited chromosome sets and one maternally inherited chromosome set, while maternal excess F1 triploid plants have one paternally inherited chromosome set and two maternally inherited chromosome sets. Notably, the chromosome sets in both reciprocal F1 triploids are genetically identical but can epigenetically differ (e.g. due to genomic imprinting or other epigenetic effects arising during maternal versus paternal chromosome transmission) with concomitant effects on parental genome dosage ratio in F1 cells and tissues.

In both stress and stress-free media, paternal-excess F1 triploid plants are larger than maternal-excess F1 triploid plants, as measured for both above- and below-ground biomass (electronic supplementary material, tables S2 and S3). For three accessions, there is a difference in salt stress response between reciprocal F1 triploid plants, indicating that there is a parent-of-origin dependent genome dosage effect on salt stress tolerance in these accessions (figure 3). In the case of L*er*-0, maternal-excess triploid plants showed a significantly lower above-ground (shoot) biomass reduction than paternal-excess triploid plants. In the case of Col-0, the below-ground (root) biomass reduction was lower in paternal-excess triploid plants than in maternal-excess triploid plants. The Wilna accession is the only case where maternal-excess triploid plants displayed a lower above- and below-ground biomass reduction than the genetically isogenic paternal-excess triploid plants. For all other accessions, the reciprocal F1 triploid pairs presented similar above- and below-ground biomass reduction rates under salt stress. On average the biomass in reciprocal F1 triploid plants is reduced by approximately 50% and below-ground biomass is reduced by approximately 75%.

## 4. Discussion

Saline conditions impose physiological constraints on plants through, first, oxidative stress, and second, ionic stress [33]. Salt accumulating in the substrate surrounding the roots inhibits the capacity of roots to take up water (an osmotic effect). Lower water availability at this stage will lead to reduced plant growth, as has been shown for *A. thaliana* [34], rice [35], maize [36,37] and barley [38]. Plant roots use the ionic composition of the substrate in which they are grown for turgor recovery, and thus can soon begin to take in water after the initial osmotic stress [39]. However, this water contains very high Na^+^ and Cl^−^ concentrations leading to the second major physiological constraint on growth: ionic stress. Na^+^ and K^+^ ions possess similar physico-chemical properties: Na^+^ can compete with K^+^ for important binding sites within the cell, impairing enzyme activity [39,40]. In addition, Na^+^ and Cl^−^ ions can accumulate in the cell wall causing cell dehydration [41]. The inability to compartmentalize/exclude harmful ions inside the cell (e.g. in the vacuole) inhibits regular cell function and leads to cell death from either toxicity or dehydration [42]. This internal injury inhibits new leaf growth, reduces overall plant photosynthesis and thus reduces the supply of carbohydrates to new cells [33]. Plants have evolved different ionic stress tolerance mechanisms, which can vary across species, and may depend on local environmental conditions as well as the length of salinity exposure. These are (i) the ability of roots to recognize Na^+^ ions and exclude them from accumulating within the plant [43–46] and (ii) tissue tolerance of Na^+^ and Cl^−^ ions through compartmentalization [40,47–50].

### 4.1. *Arabidopsis thaliana* accessions display variation in tolerance to low concentrations of NaCl but not high NaCl concentrations

*Arabidopsis thaliana* accessions which are closely genetically related display genetic variation for phenotypic characteristics, for example flowering time [51]. At low concentrations of NaCl, there were notable variations between the genetically different accessions used in this study. Four accessions (Bur-0, Col-0, T910 and Wilna) display normal or above-normal above-ground biomass accumulation at 50 mM NaCl, while others (L*er*-0, Sorbo and TAL07) display approximately 50% reduction in above-ground biomass accumulation at low NaCl concentrations (figure 1).

Previous work has demonstrated significant differences between *A. thaliana* accessions for NaCl tolerance, as measured using days-to-death [52] and leaf rosette area coupled with electrolyte leakage [53]. Our experiments did not identify a large variation in NaCl tolerance between accessions at increasingly high-stress NaCl concentrations (figure 1), even though there is some overlap with the accessions used in our study and the previous studies. However, the previous experiments used higher concentrations of NaCl than those used in our study, both in soil and in artificial growth media *in vitro* experiments.

### 4.2. Different accessions of *A. thaliana* display parent-of-origin independent and dependent genome dosage effects on salt stress tolerance

Natural and induced variations in ploidy within *Arabidopsis* populations and other crop plants can affect abiotic stress response [27,54,55]. The nucleotypic effects of polyploidy and endopolyploidy on cell volume may alter cell and tissue functions, impacting the response of plants to the environment independently of genotypic effects [56]. These nucleotypic effects can lead to an increased intracellular storage space which could be beneficial under water-limited conditions, enhancing plant water storage capacity [56]. Indeed, in environments with chronic drought stress, larger cell sizes resulting from whole-genome duplication seem to be favoured. Species in arid regions experiencing chronic drought stress often exhibit polyploidy or endopolyploidy, with polyploids showing increased drought tolerance [25,26,28,57–63]. However, the benefits of polyploidy come with costs, including increased demand for nitrogen (N) and phosphorus (P) to support elevated DNA and RNA synthesis, potentially limiting growth rates [64]. Moreover, despite the augmented cell size from genome duplication enhancing plant storage capacity and increasing tolerance to resource limitations, it may incur biomechanical costs due to reduced cell wall per unit tissue volume [65,66]. In addition to cell size, other cellular and molecular mechanisms have also been linked to the increased tolerance to abiotic stress in polyploids [23,28,55]. For instance, in tetraploid *Arabidopsis*, decreased transpiration rate, alterations in stomatal density, stomatal closure, ABA signalling and ROS homeostasis have been reported [28,67].

**Figure 4.**
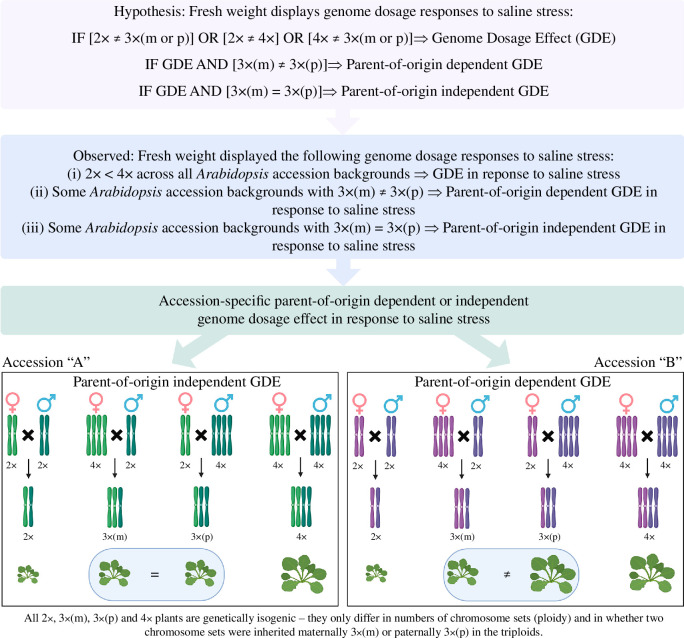
Summary of findings regarding the accession-specific parent-of-origin dependent and independent genome dosage effect in *Arabidopsis*. Created with https://www.biorender.com/.

We have previously demonstrated that *A. thaliana* tetraploid plants accumulate more above-ground biomass than their diploid equivalents [68], indicating a genome dosage effect. Likewise, paternal-excess F1 triploid plants can also accumulate more above-ground biomass than their diploid equivalent, as well as maternal-excess F1 triploid plants [68]. In this study, we do not only observe genome dosage effects but also accession-specific parent-of-origin dependent and independent genome dosage effects on salt stress tolerance (figure 4). We reveal that in some genetic backgrounds (accessions) there are parent-of-origin independent genome dosage effects on salt stress tolerance that are not evident in other genetic backgrounds (figure 3). Indeed, 5 of the 10 accessions tested (i.e. Col-0, TAL07, Wilna, Zurich and L*er*-0) displayed significant parent-of-origin independent genome dosage effects on salt stress tolerance where diploids were more stress-tolerant than both of the reciprocal triploids (figure 3). In addition, in 3 out of 10 accessions (i.e. Col-0, L*er*-0 and TAL07) the tetraploid plants showed higher stress tolerance than both of the reciprocal triploids (figure 3). If the genome dosage effects on salt tolerance we have identified were linear, we should expect that the salt tolerance value will be either 2× > 3× > 4× or 2× < 3× < 4×. However, there are no cases where such trends are evident. Instead, our results indicate that in five genetic backgrounds, the salt tolerance of both isogenic reciprocal triploid lines is lower than both the diploid and tetraploid parental lines. For Col-0, TAL07 and L*er*-0, the finding that diploid and tetraploid plants perform better under salt stress tolerance than triploid plants is strongly suggestive of a parental-genome dosage balance effect on salt stress tolerance, where plants with an equal parental contribution of chromosome sets (i.e. 2× and 4×) perform better than those with unequal parental genome dosage.

In addition, we demonstrate that there are also parent-of-origin dependent genome dosage effects on salt stress that are accession-specific. Importantly, the reciprocal triploid lines are genetically identical at the nuclear level differing only in whether the two chromosome sets in the triploid plant have been inherited paternally (via pollen) or maternally (via ovule). Any differences observed in salt stress tolerance between the maternal-excess triploid and the paternal-excess triploid are likely to have an epigenetic basis, either due to parent-of-origin specific epigenetic marks and/or parent-of-origin specific dosage-dependent factors. In this study, we have identified such parent-of-origin dependent genome dosage effects on salt tolerance in three genetic backgrounds (figure 3). Such parent-of-origin dependent genome dosage effects on salt tolerance between the reciprocal triploid plants could be due to a different distribution of epigenetic marks at the nuclear genome level (in the embryo or endosperm) [69–71], cytoplasmic differences between diploid and tetraploid maternal parents [56], or dosage-dependent factors in the maternal seed coat that differ between diploid and tetraploid maternal parents [10,72,73].

Importantly, the genome dosage effects between diploids and tetraploids, and the reciprocal F1 triploid pairs, are occurring in plants that are genetically identical (isogenic), apart from their differential genome dosage or whether their chromosome sets are maternally derived (madumnal) or paternally derived (padumnal). Hence, all such accession-specific genome dosage effects are probably epigenetic in nature as they do not involve any changes in DNA sequence [69–71].

## 5. Conclusion

The effects of soil salinization are greatest in arid and semi-arid agricultural settings. Interventions to overcome soil salinization impacts on crops can be either agronomic (e.g. drainage management) or biological (breeding crops for enhanced salt tolerance) [74]. Ploidy manipulations are widely used in crop improvement, where some studies suggest abiotic stress tolerance may be influenced by genome dosage. In this work, by using the model plant species *A. thaliana*, we have not only corroborated that genome dosage strongly affects salt stress tolerance in *A. thaliana* but also demonstrated that the nature of this genome dosage effect on salt tolerance can be parent-of-origin dependent or independent in an accession-specific manner. Hence, our study unveils new opportunities for considering epigenetic genome dosage effects in crop plants to boost their resistance to salt stress.

## Data Availability

The ploidy level of diploid, tetraploid and reciprocal triploid plants is shown in electronic supplementary material, S1 [75]. All fresh weight measurements recorded in this experiment are available in electronic supplementary material, S2.
